# What Makes You Generous? The Influence of Rural and Urban Rearing on Social Discounting in China

**DOI:** 10.1371/journal.pone.0133078

**Published:** 2015-07-14

**Authors:** Qingguo Ma, Guanxiong Pei, Jia Jin

**Affiliations:** 1 School of Management, Zhejiang University, Hangzhou, China; 2 Neuromanagement Lab, Zhejiang University, Hangzhou, China; 3 Business School, Ningbo University, Ningbo, China; University of Chicago, UNITED STATES

## Abstract

An individual’s willingness to share resources declines as the social distance between the decision maker and the recipient increases, which is known as social discounting. This social-distance-dependent prosocial behavior is likely to be influenced by the region in which individuals were raised. Based on previous studies on social discounting, this research focuses on the differing social distance-dependent prosocial behaviors between rural- and urban-reared participants in China. Our data showed that both groups’ behaviors conform to the social discounting function and fit the hyperbolic model, as reported in previous studies about social discounting. Interestingly, individuals who were raised in rural areas yielded a smaller discount rate than those who were raised in urban areas, which indicated that a rural upbringing produced people who were more generous than those with an urban upbringing. Furthermore, this distinct type of generosity occurred notably among individuals with greater social distance, such as strangers or distant acquaintances. The reason may be due to the difference in dominant culture, production mode and lifestyle between rural and urban people.

## Introduction

Humans are social beings, especially those who are more likely to display what psychologists call prosocial and altruistic behavior, such as assisting others and demonstrating generosity. During the last decades, social decision making has gained much attention. For example, it has been suggested that humans tend to be more willing to share goods and resources with those to whom they feel closer [1–3]. Therefore, people are more generous to those whom they feel closer in their decision making about finances or other resources. This decrease in generosity as a function of social distance is termed *social discounting* [4–9].

This concept of social discounting was first clearly presented by Jones and Rachlin in 2006. They also proposed that a hyperbolic function can describe social discounting, as in the cases of time and probability discounting [4]. Their research showed that generosity decreased across social distance in a non-constant, hyperbolic way that could be described by the following equation:

$$v=\frac{V}{\left(1+kD\right)}$$

where v represents the discounted value, which is the amount of willingness to forgo money to be generous towards a person at a given social distance, while D represents the social distance. The parameter V refers to the undiscounted value that determines the height of the function without affecting its shape. The degree of discounting (k) refers to the discount rate, i.e., the steepness and asymmetry of the decrease in generosity across social distance [4]. This equation shows that social discounting is defined by the adjustment between selfish and altruistic motives as a function of how much the decision maker cares about the interaction partner.

In their following studies, Jones and Rachlin investigated the impact factors that could influence the discount rate, such as total amount of reward money and decision context. They found that the degree of social discounting was an increasing function of reward magnitude by examining the discount degree as the reward money varied from a few dollars to tens of dollars and even to thousands of dollars [10]. Furthermore, culture-specific differences in social distance-dependent levels of generosity were investigated. Strombach et al. [11] found that German participants demonstrated a more pronounced decline in generosity toward close social distances in contrast to Chinese participants. Ito, Saeki, & Green [12] compared social discounting rates between Japanese and American college students. Discount rates estimated by a hyperbolic function were higher among the Japanese students than that among the American students. Moreover, they also found participants who chose the sharing option in a one-shot dilemma game showed lower discount rates. Their results suggest that discount rates reflect a cultural difference as well as a degree of selfishness.

The research described above indicated that the degree of social discounting was not constant but varied as the decision condition changed. In this study, we also considered factors that could influence the degree of social discounting. We believe that the region in which individuals are brought up could be a very important factor because history, economy, geography, lifestyle and culture differ between rural and urban residents. The data from previous studies on differing prosocial behavior between rural and urban areas suggest that a prosocial response is more likely to occur in rural communities than in urban communities [13]. Putnam found that people in rural areas, compared to people in urban areas, are more likely to volunteer, work on community projects, come to the aid of a stranger, and donate blood [14]. This evidence showed that rural residents tend to be more prosocial, especially toward strangers, than urban residents.

In China, the rural community is regarded as an acquaintance society, a concept that was first introduced by Xiaotong Fei, the founding father of Chinese sociology. In a traditional Chinese rural society with low population flow, people consistently maintain well-connected networks. Unfamiliar people inevitably become familiar, and outgroup members constantly become ingroup members [15]. In China, such connections are called the guanxi net, which is a system of gifts and favors in which obligation and indebtedness are engendered and in which there is no time limit on repayment [16].

With economic development, Chinese urban society saw a rapid increase in population, and the culture, mode of production and lifestyle in urban areas experienced great changes, while those in the rural areas continued to depend mainly on agriculture. City dwellers became gradually adjusted to handling affairs according to rules, contracts and regulations, and reliance on guanxi and ingroup connections decreased.

Similarly, urban areas were also exposed to foreign cultures, while the rural areas preserved the traditional Chinese collectivist culture. Researchers theorized that when two or more cultures exist together, people tend to be allowed more individuality [17].

From the above-mentioned information, it can be speculated that region could be an important moderating variable in social decision making. The discount function depends on how the participant organizes his or her environment. The goal of our study is to investigate the influence of region on a specific type of social decision making that depends on social distance. We designed the experiment in accordance with Jones and Rachlin’s [4] study and divided the participants into two groups, rural and urban, according to the areas in which they were raised. We hypothesized that generosity was a decreasing function of social distance, independent of the region where the participants were raised, which meant that generosity to others would be influenced by social distance and fit the hyperbolic model well in both groups. Second, region would influence the shape of the social discount curve. Participants who were brought up in rural areas may be more generous than those brought up in urban areas, which would be reflected in a lower discount rate due to these individuals being more prosocial than urban individuals. Third, we theorized that the difference in generosity between rural and urban people was greater when paired with recipients with greater social distance, such as strangers or others who were not considered close to the decision maker.

## Methods

### Participants

A total of 103 (55 male) healthy undergraduates were enrolled from Zhejiang University through the online community. 47 (25 male) grew up in the countryside, and the other 56 (30 male) participants were raised in urban areas. Data from 5 (2 rural and 3 urban) participants were eliminated from the study because they misunderstood the pre-experiment or some criteria were not satisfied, resulting in 98 valid participants for final data analysis. The study has been conducted with the ethical approval of the Internal Review Board of Neuromanagement Lab of Zhejiang University. The privacy and rights of the participants were observed. Each individual provided written informed consent. All participants received monetary compensation for their time.

### Experiment design and procedure

The experimental procedure was nearly identical to the one used in Strombach et al. [11]. It consisted of three parts: the pre-experiment, the formal experiment and the follow-up questionnaire. The experiment was presented using E-Prime 2.0 software package (Psychology Software Tools, Pittsburgh, PA, USA). In the pre-experiment, participants were asked to score the perceived closeness of fourteen specific people in their social environment such as their mother, father, friends, neighbors, strangers and so on, from 1 to 100 (1 indicating the closest-possible relationship and 100 indicating the most distant). If one of the people did not exist in the social environment of the subject (e.g., partner or a deceased relative), this task was skipped. The task can be regarded as a manipulation check to control for fundamental differences in self-representation. The ratings should therefore not contradict the allocated social distances in the following social discounting experiment; if so, the participant would be eliminated.

During the formal experiment, the trials were randomly presented. Social distance was represented on a ratio scale and transformed into a scale consisting of 100 icons. The first icon on the left represented the person himself or herself. The icon next to it (social distance 1) represented the person within the participant’s social environment to whom the participant felt closest, e.g., his/her mother. The icon on the opposite end of the scale (social distance 100) represented a person considered the most socially distant, a person the participant did not care about at all but also for whom the participant had no negative feelings—for instance, a random stranger.

**Fig 1:**
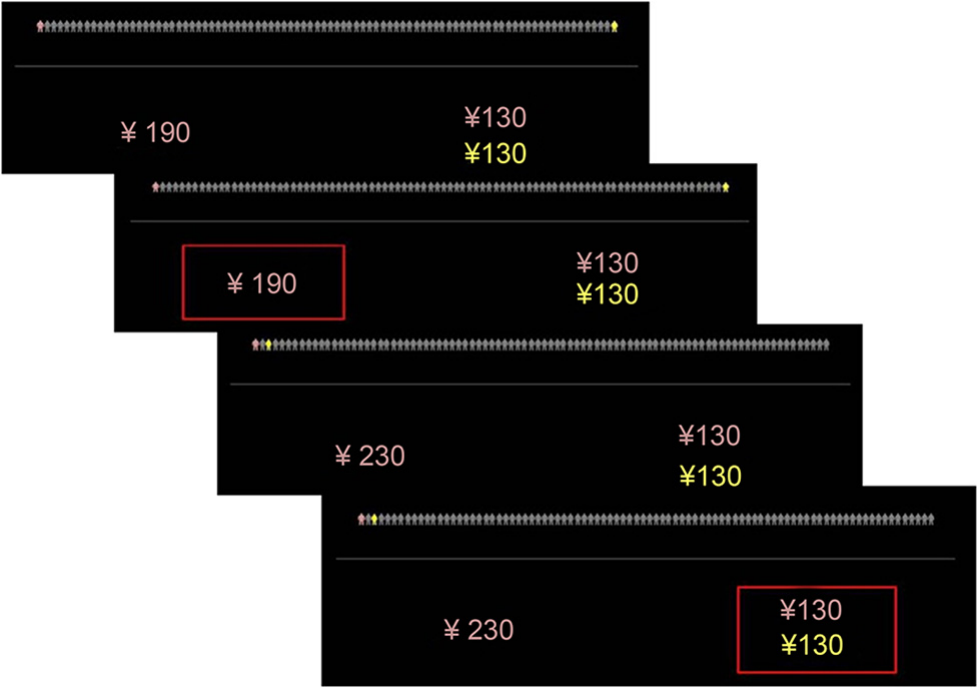
Example of the social discounting experiment. First, the social distance information and a generous and selfish reward were presented. The decision result was then shown.

As in Jones and Rachlin’s experiment, decisions had to be made for the following seven social distances: 1, 2, 5, 10, 20, 50, and 100 [4,10]. The person at the given social distance for that trial was marked yellow. The participant was asked to imagine a real person as a representative of that specific social distance. In each trial, the participant had to choose between a selfish and a generous option, yielding either a large reward for the participant only or a smaller reward for the participant also accompanied by a reward to another individual at a certain social distance as presented in Fig 1. The color-coded numbers beneath the 100 icons indicated the recipient and the magnitude of that reward. The selfish option varied between 130 and 290 yuan in increments of 20 yuan across trial repetitions. The generous option was a fixed amount of 130 yuan for the participant and 130 yuan for the interaction partner. Therefore, each participant was required to perform 63 trials (7 types of social distance×9 monetary amounts for the selfish option).

In the subsequent task, participants were asked to complete a questionnaire naming and describing his or her relationships to the persons used as representatives for each of the seven social distances, along with personal information, such as sex, age and the region in which they were raised. Participants received a 10 Yuan participation fee. In addition, at the end of the experiment, one trial within the experiment was randomly chosen, and 5% of the real decision value was paid as part of the participation fee. If a selfish option was selected, the participant received between 16.5 yuan and 24.5 yuan, depending on the trial chosen. If the decision was generous, the participant received 16.5 yuan and the other person 6.5 yuan. Participants were asked to record the address of the virtual interaction partner in that trial, which all participants were able to do. The interaction partners received their rewards by mail. Participants also had the option of donating the interaction partner’s money to a charity instead (China Youth Development Foundation). Information about this option was only given at the end and thus could not influence our participants’ decisions. The experiment did not involve deception and was performed in an incentive-compatible way; thus, it met the standards for economic research [18,19].

### Data analysis

In the pre-experiment section, an independent sample t-test was performed to compare the perceived distance to each specific person. Participants who did not understand this part were dismissed—for example, if someone considered 1 to be the most socially distant and 100 the least socially distant. For the social discounting experiment, at each level of social distance, the point at which the participant was indifferent between the two options was determined by titrating the selfish reward magnitude, which revealed how much money the individual was willing to forgo to give a reward to the specific person [4]. Logistic regression was used to determine the point of indifference, i.e., the point at which the statistical probability of answering generously and selfishly was 50%. If a participant decided exclusively generously or exclusively selfishly at a particular social distance level, the crossover points were assumed to be 300 and 120 yuan, respectively. The amount of money forgone was calculated by the indifference point, equal to the indifference point minus 130, which was the amount of money the participant would receive if he chose the generous answer. A standard hyperbolic model was fitted individually to the resulting data points in the two regions [4]. Additionally, to investigate differences across regions, the Mann-Whitney U test was conducted to investigate the difference of k and V and the amount of money forgone at each social distance. Furthermore, two related sample tests were also conducted to analyze the generosity decay between adjacent distances by using a Wilcoxon test in each group separately.

## Results

### Regional Differences in Self-Construal

**Fig 2:**
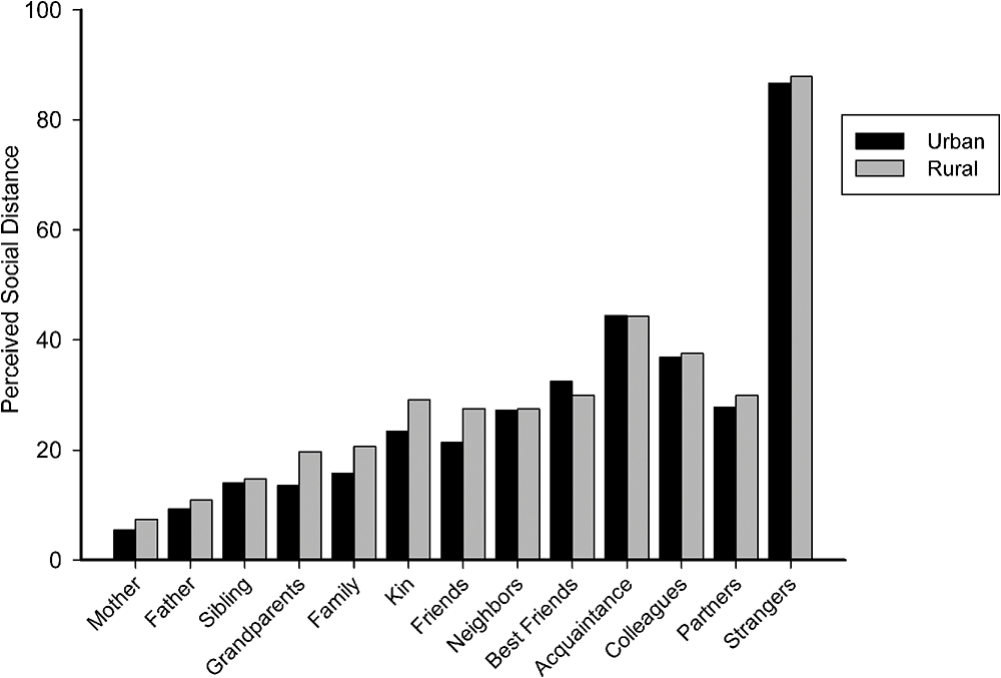
Difference of perceived closeness to a specific person between rural and urban groups. Lower numbers indicate greater closeness.

An independent sample t-test was performed to compare the perceived distance to each person listed in pre-experiment section. There was no significant difference in the rating of closeness between the two region groups for each relation, as shown in Fig 2 and Table 1.

**Table 1:** The difference of self-construal between urban(U) and rural(R) groups.

	*t-Value*	*p-Value*	*Mean*	*Std*. *Deviation*
**Mother**	-1.525	.130	U = 5.4717; R = 7.4000	U = 5.70965; R = 6.80708
**Father**	-.726	.470	U = 9.2692; R = 10.9111	U = 12.41460; R = 9.36585
**Sibling**	-.286	.776	U = 14.0189; R = 14.7778	U = 13.87234; R = 12.11102
**Grandparents**	-1.812	.064	U = 13.5283; R = 19.7111	U = 12.96831; R = 19.52693
**Family**	-1.611	.111	U = 15.7308; R = 20.6136	U = 14.36378; R = 15.29891
**Kin (Other Relatives)**	-1.677	.097	U = 23.4340; R = 29.1556	U = 15.01219; R = 18.75555
**Friends**	-1.280	.204	U = 21.3962; R = 27.4889	U = 21.63361; R = 25.48406
**Neighbors**	-.056	.956	U = 27.2308; R = 27.4444	U = 18.44931; R = 19.14722
**Best Friend**	.466	.642	U = 32.4528; R = 29.8889	U = 28.44736; R = 25.50599
**Acquaintances**	.010	.992	U = 44.3774; R = 44.3333	U = 21.43167; R = 21.38925
**Colleagues**	-.192	.848	U = 36.8868; R = 37.5556	U = 17.71099; R = 16.57018
**Partners**	-.478	.634	U = 27.7826; R = 29.8947	U = 20.23519; R = 20.03077
**Strangers**	-.374	.709	U = 86.6604; R = 87.8667	U = 15.71861; R = 16.16759

### Social Discount Function

**Fig 3:**
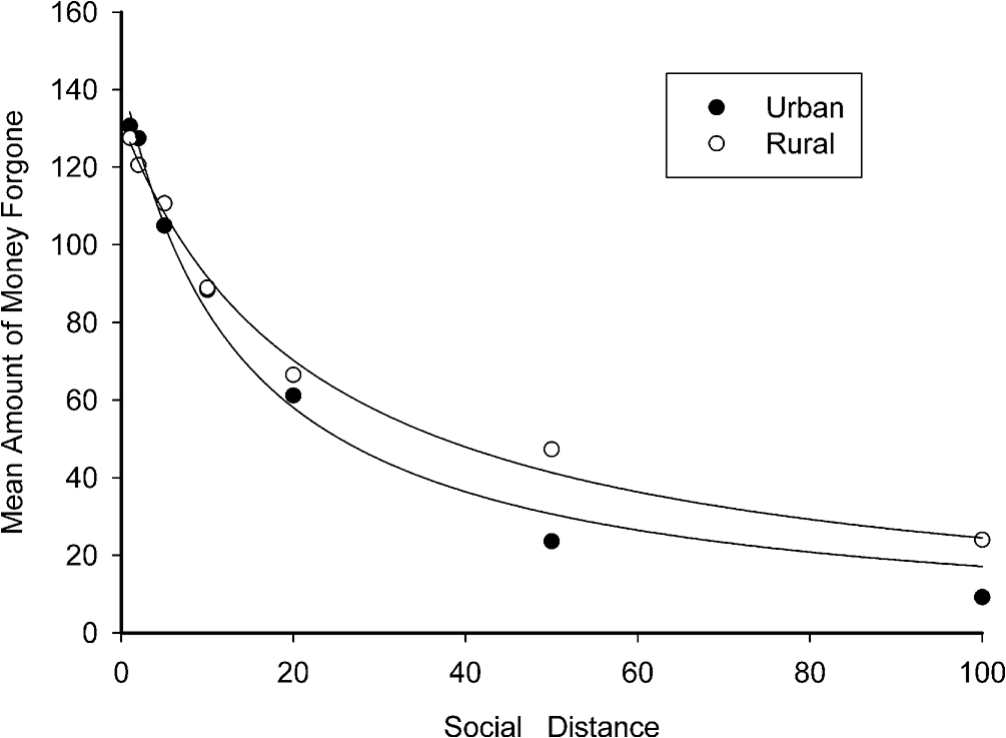
Fitting of the Hyperbolic Discount Function for both the two regions.

Results showed that participants from both regional backgrounds were less willing to forego a reward and be generous to a person as social distance increased, which replicated prior research findings [4,5,10]. A standard hyperbolic model [20] was fitted to the mean value of the amount forgone separately for each group, indicating a good fit for the rural (X² = .5925, R²_corr_ = .9931) and for the urban data (X² = 3.6397, R²_corr_ = .9912), as summarized in Table 2. Fig 3 presents the mean amount forgone and the hyperbolic discount function fitted curve of the two groups.

**Table 2:** Social Discount Parameters of urban and rural groups.

*Model*	*Region*	*Mean Fit*	*Fitted Parameters*
**Hyperbolic Model**	***urban***	r² = .9912; X² = 3.6397	k = 0.07410; V = 144.2071
**Hyperbolic Model**	***rural***	r^2^ = .9931; X^2^ = .5925	k = 0.04390; V = 132.0377

The function had two free parameters, V and k. V represented the undiscounted value and k corresponded to the individual discount rate [4,21]. We found significant differences in k across regions (U = 856.5, Z = -2.395, p = .017); specifically, the participants with a rural upbringing (mean value: 0.1102) had smaller k values than urban-reared participants(mean value: 0.1894). However, there was no difference in V between rural and urban participants (U = 1026.0, Z = -1.183, p = .237). Thus, the rural participants had a smaller discount rate than those from urban areas, which indicated that their generosity levels decay more slowly across social distance.

**Fig 4:**
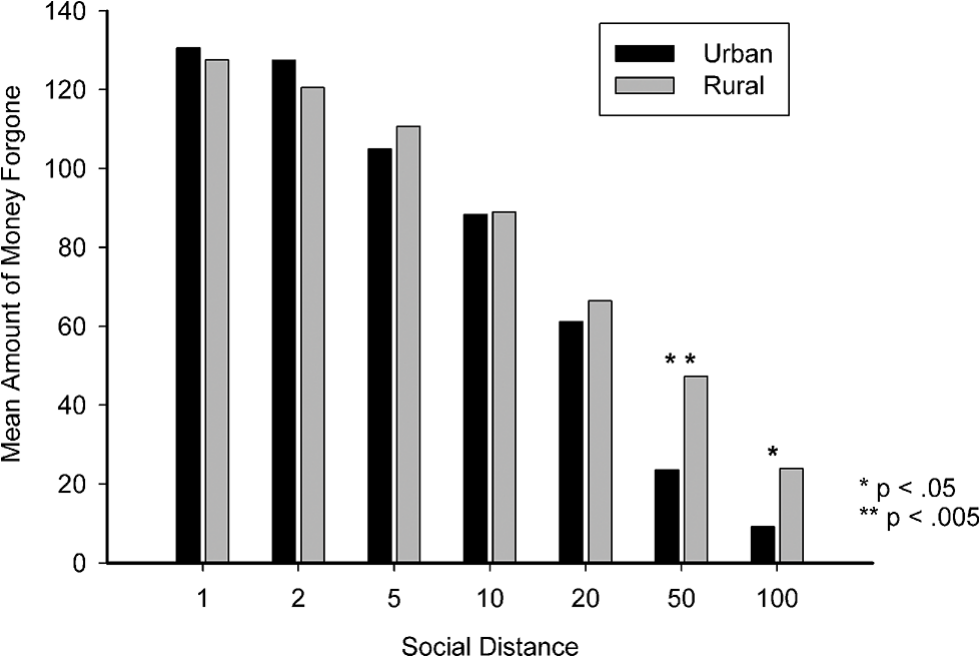
Mean amount of money forgone per social distance in rural and urban regions.

Moreover, we also found effects of region of upbringing when comparing levels of generosity per social distance. Several Mann-Whitney U tests were conducted due to inhomogeneous variances. As summarized in Table 3, there were significant differences between the two groups at social distance 50 (U = 716.50, Z = -3.465, p = .001) and 100 (U = 866.00, Z = -2.314, p = .021). Compared with urban participants, the rural participants had greater mean amounts of forgone money to be generous to the interaction partner in these two distances (Fig 4).

**Table 3:** Generosity per social distance between urban(U) and rural(R) groups.

*Social Distance*	*z-Value*	*p-Value*	*Average Rank*	*Mean*	*Std*. *Deviation*
**1**	-.399	.690	U = 48.51; R = 50.67	U = 130.62; R = 127.56	U = 28. 899; R = 38.364
**2**	-.632	.528	U = 51.09; R = 47.62	U = 127.45; R = 120.53	U = 37.329; R = 45.158
**5**	-.665	.506	U = 47.78; R = 51.52	U = 104.92; R = 110.64	U = 48.166; R = 42.393
**10**	-.215	.830	U = 48.93; R = 50.17	U = 88.38; R = 88.96	U = 51.754; R = 45.930
**20**	-.642	.521	U = 47.81; R = 51.49	U = 61.17; R = 66.49	U = 50.906; R = 46.430
**50**	-3.465	.001	U = 40.52; R = 60.08	U = 23.60; R = 47.31	U = 27.208; R = 36.475
**100**	-2.314	.021	U = 43.15; R = 55.76	U = 9.19; R = 24.00	U = 17.881; R = 35.213

### Regional difference in generosity decay

Two related sample tests were conducted to analyze the difference between adjacent distances using a Wilcoxon test. The amount of money forgone was compared across the social distances per region. As shown in Table 4, for the urban sample, the results indicated a significant drop in generosity between each pair of adjacent social distances, except at social distance 1 (M = 130.62, SD = 28.899) and social distance 2 (M = 127.45, SD = 37.329, z = -0.058, p = .954). By contrast, in the rural sample, the difference in generosity between social distance 1 (M = 127.56, SD = 38.364) and social distance 2 (M = 120.53, SD = 45.158) was not pronounced (z = -1.559, p = .119), nor was the difference between social distance 2 and social distance 5 (M = 110.64, SD = 42.393, z = -1.022, p = .307). However, the drop between other adjacent social distances was significant. This result showed that urban individuals displayed a strong decrease in generosity at close social distances, while rural participants showed a less profound effect, indicating an unclear differentiation between the self and people considered close to the participant.

**Table 4:** The degree of generosity decay of the two regions.

Region	Social Distance	*z-Value*	*p-Value*
**Urban**	Distance 1 vs. Distance 2	-0.058	.954
	Distance 2 vs. Distance 5	-3.680	.000
	Distance 5 vs. Distance 10	-3.673	.000
	Distance 10 vs. Distance 20	-3.613	.000
	Distance 20 vs. Distance 50	-4.888	.000
	Distance 50 vs. Distance 100	-3.987	.000
**Rural**	Distance 1 vs. Distance 2	-1.559	.119
	Distance 2 vs. Distance 5	-1.022	.307
	Distance 5 vs. Distance 10	-3.361	.001
	Distance 10 vs. Distance 20	-3.410	.001
	Distance 20 vs. Distance 50	-3.037	.002
	Distance 50 vs. Distance 100	-4.073	.000

## Discussion

The objective of this study was to investigate regional differences between rural and urban people in social discounting. In general, we hypothesized that participants would be less willing to forego money and be less generous to specific people with increasing social distance in both the urban and rural groups, and the hyperbolic model would reflect this. We also expected to find regional differences in the shape of the social discount function, which was reflected in the different discount rates. Furthermore, we also believed the generosity difference was largely occurring with distant relationships.

Our results confirmed these hypotheses. Participants’ behaviors followed the social discounting concept regardless of urban or rural upbringing. Further analyses also revealed that the region had an influence on social-distance-dependent levels of generosity. Participants who were brought up in rural areas had lower degrees of discounting, which illustrated that rural-reared people were more generous to others than urban-reared participants. The study showed that rural-reared participants perceived sharing a reward as preferable to keeping a reward for oneself. Thus, sharing seemed to have been a high priority in rural participants’ decision making process. In contrast, urban participants were less willing to forgo money unconditionally. Moreover, by comparing the difference in each social distance, we found significant differences of generosity levels between the two groups at social distance 50 and 100. Rural participants expressed a significantly higher level of generosity in general, but especially at social distance 50 and 100.

There are several reasons for this. First, we believed that in urban areas of China, the dominant culture leans toward individualism compared with the culture in rural areas, which is more inclined toward collectivism. Previous studies have demonstrated a strong correlation between increased economic development and individualism. More economically developed regions are more individualistic, and less developed regions are more collectivistic [22, 23]. Additionally, rural residents have little opportunity to make contact with other cultures, as well as traditional Chinese culture tends toward collectivism. Furthermore, rural residents may experience more pressure to conform traditional culture than people from urban areas [24–26]. In contrast, urban residents in China are exposed to several foreign cultures and experience less pressure to conform to a specific traditional culture. It also has been found that when two or more cultures coexist, people tend to be allowed more individuality [17]. Due to a less-developed economy and limited social and cultural diversity relative to urban areas, rural culture leans toward collectivist compared with urban culture. Individualistic cultures value independence, autonomy, and self-reliance and individuals personal goals take priority while collectivistic cultures differ because they value interdependence, cooperation, and social harmony [27, 28]. Therefore, people in collectivist cultures make weaker distinctions between socially close and distant people, leading to a generally higher proportion of prosocial behaviors that benefit interaction partners [29]. Therefore, the degree of difference with regard to prosocial tendencies is large between Chinese rural and urban areas.

Second, in rural areas, farming still plays a significant role in everyday life. Farmers are required to cooperate to run a successful farm [17]. This necessity might therefore change rural individuals’ perception of strangers. They might perceive a stranger not as a stranger but as a part of their social environment, leading to a higher degree of generosity towards people in general. However, for the urban participants, this perception of strangers may not exist.

Third, research over the years has indicated that rural communities have higher levels of social integration and attachment than urban communities. In traditional Chinese rural society (acquaintance society) with low population flow, people consistently maintain a well-connected network. For rural people, because of the lag in economic development, translation from the rule of man to the rule of law is a distant objective. Reliance on guanxi is still considered essential. Guanxi, as an important social resource, is still essential to successfully interacting in virtually all spheres of social life and to enjoy a high quality of life. However, city dwellers have become used to handling their affairs according to rules, contracts and regulations with the development of the market economy. The relationship between guanxi and increased quality of life has, therefore, weakened. To avoid continuous distraction, people become less involved in the affairs of others. This leads urban participants to exhibit less concern toward others, especially those with greater social distance. Sampson also demonstrated that urbanization had a negative association with local friendships and attachment at the collective level [30]. Living in a large city causes residents to develop a reduced sensitivity to their social environment. Compared with urban communities, rural communities have higher levels of social integration, and rural people demonstrate more prosocial behaviors to consciously cultivate and maintain guanxi. They are inclined to reduce social distance with strangers and behave more generously.

Moreover, we also found that urban individuals showed a marked decrease in generosity at social distances of 2 and 5, while rural participants showed this type of generosity decay at social distances of 5 and 10, which showed that the generosity decay of urban participants was stronger than that of rural participants with regard to close social relationships. The reason for this difference may be that rural people are more traditional, family-oriented and collectivistic compared with urban people. In traditional Chinese culture, the individual is subordinated to the family. Family members are educated to restrain their individualism to maintain harmony within the family. The family-child relationship in Confucianism models a reciprocal relationship in which children serve their parents with filial piety and submission, and parents treat their children with kindness and care [31]. Even when children become economically independent, they are still subject to the influence of their parents. Children are responsible for their elderly parents. Therefore, compared with urban people, rural people are more traditional, family-oriented and more generous to persons in close social relationships, especially their parents.

Additionally, although the participants in our study were all enrolled from a university in the city, participants in the rural groups has lived in the city for one to four years. Research suggested that people from rural areas tend to maintain their original attitudes and behavior even when they move from a rural area to an urban area, and vice versa. Researchers believe that one’s place of origin is a better indicator of how one behaves. Furthermore, they also suggest that rural residents are more disposed to maintaining existing views and are more hesitant to change compared to their urban counterparts [32]. Therefore, we believe that the participants’ one to four years’ university lives would not measurably influence their behavior, and our result confirmed this hypothesis. Studying university students has the additional advantage that current differences in environmental variables cannot explain the results, as the environment in which the students live is highly comparable.

In summary, region plays a pivotal role in social decision making, and research should take these differences into account when studying social decision making. Our results showed that rural individuals tend to be more prosocial and more generous to others than urban individuals, especially to those with greater social distance. Additionally, their decay of generosity in close social relationships also differed, as the rural group’s initial decay appeared sharply between a distance of 5 and 10, while the urban group showed an initial decay between a distance of 2 and 5. These social-distance-dependent differences in generosity between the two groups of participants were explained in terms of their differences on dominant social culture, production modes and lifestyle.

## Supporting Information

S1 File Zip file containing raw experimental data. (ZIP)
